# Dengue virus and infertility: effects on reproductive health and outcomes

**DOI:** 10.5935/1518-0557.20250030

**Published:** 2025

**Authors:** Victória Campos Dornelles, Gabriela Barella Schmidt, Ana Luiza Berwanger, Sophia Abur Said, Laura Randon Chapochnicoff, Carolina Comissoli Fernandes, Marta Ribeiro Hentschke, Alvaro Petracco, Mariangela Badalotti

**Affiliations:** 1 School of Medicine, Pontifical Catholic University of Rio Grande do Sul (PUCRS), Porto Alegre, Brazil; 2 Post graduate program of Medical Science, Federal University of Rio Grande do Sul (UFRGS), Porto Alegre, Brazil; 3 Fertilitat - Medical Reproductive Center - Porto Alegre, Brazil

**Keywords:** dengue, infertility, viral infection

## Abstract

Brazil has been facing periods of dengue epidemics for more than 40 years. Recently, the disease showed increasing numbers of diagnoses. This scenario is alarming in terms of public health, and has caused concern in specific areas of medicine, such as Assisted Reproduction. What influence does viral infection have on the success of treatments? What is the risk of acquiring the virus in the early stages of pregnancy? Various data confirm vertical transmission, although the risks are extremely low and sexual transmission of the infection seems unlikely. The aim of this article is to review the literature on the influence of Dengue infection on Assisted Reproduction treatments, with a view to advising patients who are planning to start treatment.

## INTRODUCTION

### Dengue and infertility

#### Viral factors and infertility

Viral factors are among several elements capable of affecting human fertility. In women, viral infections can cause tubal factor infertility, ectopic pregnancy, and fetal prematurity. In men, these infections can impact sperm DNA integrity, as well as sperm concentration, motility, viability, and morphology. It is estimated that up to 15% of male infertility cases are linked to viral factors (Sucato *et al.*, 2023).

#### Dengue infection

Dengue is an acute febrile, systemic, and dynamic infectious disease caused by an arbovirus from the Flaviviridae family. The primary vectors are female *Aedes aegypti* mosquitoes, and to a lesser extent, *Aedes albopictus*. These mosquitoes also transmit chikungunya and Zika. In 2023, 1,658,816 probable cases of dengue were reported in our country, primarily in the Southeast (56.41%), followed by the South (23.8%) and Midwest (11.5%) (Kularatne & Dalugama, 2022; Brazil, 2024).

#### Dengue transmission

Dengue can be transmitted vertically (transplacental) or during the perinatal period. Breastfeeding has been proposed as a route of vertical transmission of the dengue virus (DENV), with some cases described in the literature. Sexual transmission of DENV remains questionable. According to Basurko *et al.* (2009), the risk of fetal transmission is higher when infection occurs close to childbirth (Molton *et al.*, 2018; Cruzeiro & Oliveira, 2024).

#### Presence of DENV in gametes

In mosquitoes, DENV infection has been shown to significantly reduce fecundity, influencing reproduction at the genetic level and inducing specific transcriptional changes (Feitosa-Suntheimer *et al.*, 2022). However, there is currently no evidence indicating that DENV infection affects human ovaries as it does mosquito ovaries. In men, acute dengue infection has been found to impact semen characteristics, with viral RNA detectable in semen samples up to 30 days after symptom onset (Mons *et al.*, 2022). Notably, motile sperm were not infected, and the virus persisted in semen for a shorter duration compared to blood samples, suggesting that any impact on sperm may be reversible (Mons *et al.*, 2022). Further research is needed to determine if the DENV affects human ovaries and to further explore its impact on semen.

#### Influence of other viral infections on gametes and reproductive treatments

Many viruses exhibit a strong tropism for the male reproductive system, particularly the testicles. Several factors influence viral presence in semen, including viremia levels, specific viral epitopes, immune replication mechanisms, viral structural stability, specific receptors on testicular cells, and the presence of other sexually transmitted pathogens. Different viruses target distinct cell types in the reproductive tract, and cellular responses vary among species (Teixeira *et al.*, 2021). Flaviviruses can undergo vertical transmission, though this is relatively inefficient, with estimated filial infection rates ranging from 1/36 to 1/6,400. Zika virus (ZIKV) is currently the only known arbovirus linked to sexual transmission in humans, enabling viral spread into multiple body fluids, including semen (Gornet *et al.*, 2016; Li *et al.*, 2018). Infected male patients may have ZIKV RNA in their blood, saliva, and urine. ZIKV can cross the blood-testis barrier and replicate in the reproductive system (Barzon *et al.*, 2016; Brooks *et al.*, 2016). In female patients, ZIKV can be sexually transmitted and transmitted vertically, as evidenced by its presence in amniotic fluid, placenta, and fetal brain tissue of fetuses with microcephaly (Barjas-Castro *et al.*, 2016; Motta *et al.*, 2016; Swaminathan *et al.*, 2016; Washington *et al.*, 2016; Cavalcanti *et al.*, 2017; Li *et al.*, 2018).

#### Influence of viral infections on assisted reproduction

The COVID-19 pandemic presented challenges for the field of Assisted Reproductive Technology (ART) because the effects of SARS-CoV-2 on ART treatments were initially unknown. As the pandemic progressed, the American Society for Reproductive Medicine (ASRM) made recommendations for managing these risks. In general, given the hormonal, immunological, and pro-thrombotic risks of controlled ovarian stimulation, anesthesia, and surgical procedures in the context of COVID-19, treatment was recommended to be carefully adapted to each patient to minimize risk during the endemic period.

Regarding dengue, it remains unclear whether the disease affects the outcomes of ART for infertility treatment (Geber *et al.*, 2014). For women contracting dengue during ovulation induction, literature data is limited. The current recommendation is to inform the attending physician, who will assess the situation and provide guidance (Cruzeiro & Oliveira, 2024). According to the Centers for Disease Control and Prevention (CDC), dengue virus testing is not recommended for asymptomatic patients or as preconception screening. For couples in endemic areas, preventive measures are advised, including vaccination, repellent use, and elimination of standing water.

Vaccinated individuals should wait 30 days before attempting natural conception or ART treatments. Likewise, if one partner is diagnosed with dengue, a 30-day waiting period is recommended before conception attempts.

Dengue infection has been associated with increased risks of miscarriage, prematurity, and low birth weight. Depending on the stage of treatment and disease severity, ovulation induction may need to be postponed until full recovery. If ovarian stimulation has already begun, oocyte retrieval with total freezing of eggs or embryos may be the safest option (Cruzeiro & Oliveira, 2024). For men undergoing ART and testing positive for dengue, it is worth noting that fever may affect seminal quality. Additionally, ovarian hyperstimulation can exacerbate dengue symptoms by increasing dehydration and hypercoagulability.

In pregnant women diagnosed with dengue, anticoagulants and antiplatelet agents should be suspended due to bleeding risks (Cruzeiro & Oliveira, 2024).

#### Dengue vaccine and ART

The dengue vaccine, Dengvaxia (CYD-TDV), raises considerations for safety and efficacy in reproductive-age and pregnant women. It is a live attenuated tetravalent viral vaccine approved for use in endemic regions (Wong *et al.*, 2022; Malik *et al.*, 2023). Pregnant women with dengue infection face increased risks, including preterm labor and delivery. Vertical transmission from mother to infant has been reported; however, the potential for transmission of the vaccine virus remains unknown (DailyMed, 2023). No specific studies have been conducted on pregnant women, and the vaccine is contraindicated during pregnancy due to the risks associated with live attenuated vaccines (Skipetrova *et al.*, 2018; DailyMed, 2023).

Inadvertent vaccination during pregnancy has not shown increased adverse outcomes compared to controls, such as stillbirth or spontaneous abortion (Skipetrova *et al.*, 2018). In seronegative individuals, the vaccine may cause antibody-dependent enhancement (ADE), leading to severe dengue upon subsequent natural infection, which necessitates confirming prior dengue infection before vaccination (Dans *et al.*, 2018; Sridhar *et al.*, 2018; DailyMed, 2023). In animal studies, no fetal harm was observed in rabbits at human-equivalent doses, though fetal toxicities were noted in mice (DailyMed, 2023).

No direct evidence suggests that the vaccine affects gametes or ovarian function in the context of ART. However, it is generally advised to avoid live vaccines during pregnancy, ensuring that women are not pregnant at vaccination due to pregnancy-related immunosuppression (Skipetrova *et al.*, 2018; DailyMed, 2023). Thus, Dengvaxia should be administered with caution in reproductive-age women undergoing ART, ensuring they are not pregnant and have confirmed prior dengue infection. Given its relatively recent approval and anticipated broader use, further research is necessary to investigate potential effects on ART patients.

## MATERIAL AND METHODS

A descriptive-documentary review was carried out using an advanced search on the Public Medline (PubMed), Scientific Electronic Library Online (SciELO) and Latin American and Caribbean Health Sciences Literature (LILACS) database platforms.

The search was carried out in September 2024, using the keywords: “IVF,” “embryo quality,” “male factor,” “oocyte quality,” “semen,” “infertility” and “dengue virus infection,” using the Boolean operator “OR/AND” as appropriate, resulting in 42 articles in total. Scientific articles were included in the study. Articles published in English and dated within the last ten years were selected. Case reports, book chapters, letters to the editor, viewpoints, studies with hypothetical data or scenario simulations and studies that did not include any of the information expressed by the inclusion criteria were not included in the study. Studies that had not been conducted on humans were also not included.

The articles were assessed for their title and abstract, according to the inclusion criteria. The full version of the selected documents was then assessed by independent reviewers.

## DISCUSSION

The growing research on DENV epidemiology and transmission routes reveals significant implications for both public health and reproductive medicine. Studies such as Guo *et al.* (2017) and Juraska *et al.* (2018), provided insights into the geographic and genetic diversity of DENV, affecting transmission dynamics and challenging vaccine development and deployment, emphasizing the need for localized public health strategies. Hence, this diversity combined with the complex immunogenic responses to DENV highlighted in recent vaccine studies (Jackson *et al.*, 2018; Tran *et al.*, 2019; Izmirly *et al.*, 2022) that underscored the challenges faced by individuals in dengue-endemic regions, particularly those of reproductive age or undergoing ART.

Regarding these vaccine studies, phase 1 researched by Jackson *et al.* (2018) provided critical insights into the safety and effectiveness of a tetravalent dengue vaccine, supporting its role in controlling outbreaks. Tran *et al.* (2019) further confirmed the CYD-TDV vaccine’s long-term safety and benefits across various populations. Expanding on this, the study of Izmirly *et al.* (2022), examined pre-vaccination immune markers and showed how individual immune profiles can influence vaccine response, which is essential for optimizing vaccination strategies. Additionally, studies by Martínez-Vega *et al.* (2016), and Gupta *et al.* (2018), highlighted the challenges of dengue control in high-incidence areas, reinforcing the need for strong public health interventions.

Emerging hypotheses around potential non-vector transmission routes of DENV, such as sexual transmission, open new avenues of inquiry. Analogous to findings in Zika virus, that has already been demonstrated to be sexually transmitted (Hastings & Fikrig, 2017), there is preliminary evidence suggesting possible alternative transmission pathways also for DENV (Blitvich *et al.*, 2020). Such mechanisms could have implications for DENV control strategies and underscore the importance of studying its potential harm on reproductive health and possible effects on ART outcomes.

The physiological effects of acute DENV infection, which includes febrile and inflammatory responses, may exert indirect negative influences on fertility, In the context of female reproduction, febrile illnesses can disrupt menstrual cyclicity and ovulatory patterns (Blitvich *et al.*, 2020). Thus, DENV may have implications for fertility and (Paixão *et al.*, 2019) pregnancy. Although direct evidence linking DENV to ovarian or uterine dysfunction remains limited, the stress response and immune activation caused by DENV infection could temporarily reduce fertility. This hypothesis aligns with general viral impacts on reproductive function and calls for more detailed studies on the virus’s effects on ovarian health and function (Hastings & Fikrig, 2017; Paixão *et al.*, 2019).

Furthermore, pregnancy represents a state of altered immunologic balance, making pregnant women particularly vulnerable to viral infections. Cohort studies and systematic reviews, such as those by Paixão *et al.* (2016; 2019), have associated maternal dengue infection with adverse pregnancy outcomes, including miscarriage, preterm birth and low birth weight. These studies presented significant risks to both maternal and fetal health, particularly in cases of severe dengue infection (Martínez-Vega *et al.*, 2016; Paixão *et al.*, 2016; 2019), in agreement with a recently published systematic review and meta-analysis by Shabil *et al.* (2024), in which maternal dengue infection was also associated with a higher prevalence of preterm birth and low birth weight, although in these latter study results were not statistically significant (Shabil *et al.*, 2024).

In assisted reproduction, DENV poses specific challenges due to its potential immunologic effects. DENV-related inflammation and immune responses may impact gamete quality and uterine receptivity, both crucial factors for ART success. Studies such as those by Jackson *et al.* (2018) and Tran *et al.* (2019), on vaccines and immune responses have provided insights into how dengue infection might affect the success of procedures like IVF. Insights from Izmirly *et al.* (2022) on pre-vaccination immune markers and responses are particularly relevant in this context, as they suggest that individual immune profiles could modify ART outcomes in patients with DENV infection. The interaction between DENV-induced immune response and the hormonal environment needed for successful ART procedures needs further investigation, especially regarding optimal timing and immune modulation strategies for patients recovering from recent DENV infections (Nascimento *et al.*, 2017).

Encompassingly, while limited data exist on the direct effects of DENV on fertility and ART outcomes, the infection’s physiological and immunologic impacts suggest a potential for interference in reproductive outcomes.

## CONCLUSION

The collective findings from these studies highlight the multifaceted nature of dengue virus transmission and control. Current evidence indicates that mosquito-borne transmission remains the primary route, while emerging concerns about alternative pathways, such as potential sexual transmission, warrant further investigation. Moreover, advancements in vaccine development and epidemiological surveillance are essential in managing and mitigating dengue outbreaks, and much of the existing literature focuses on these areas. Future research should continue to explore these aspects to develop comprehensive strategies for global dengue prevention and control.

Overall, the interaction between dengue infection and outcomes may also be complex and is not yet fully understood. While current research provides a preliminary foundation, further studies are essential to rigorously assess the hypothetical effects of dengue infection on fertility and ART outcomes. There is still a significant gap in evidence-based guidelines for managing these cases. Combining epidemiological data, immunological insights and research on pregnancy outcomes, the clinical management of women affected by dengue virus while seeking ART could be better optimized.

## FINAL CONSIDERATIONS

While knowledge regarding DENV epidemiology and transmission mechanisms has advanced, the relationship between the infection and its reproductive outcomes lacks direct and robust data. Evidence from similar viruses, such as Zika, have already demonstrated sexual transmissibility, raising questions about the still unknown and potential alternative DENV transmission routes. These still understudied possibilities could have implications for reproductive health (Hastings & Fikrig, 2017; Blitvich *et al.*, 2020).

DENV indirect effects, including fever, inflammatory responses, and physiological stress, may temporarily affect ovarian function and menstrual cycles, potentially harming fertility. In the context of assisted reproduction, DENV’s immunologic and inflammatory effects could also alter gamete quality and uterine receptivity, factors that are essential for achieving pregnancy. The increased risks for adverse pregnancy outcomes associated with DENV infection have already been shown, such as preterm birth and low birth weight, highlighting the need for special monitoring of women of reproductive age in dengue-endemic regions (Paixão *et al.*, 2016).

Given the current gaps in literature, additional research is essential to clarify the mechanisms on which DENV might impact both spontaneous and assisted reproductive pregnancies. Understanding these associations may improve evidence-based guidelines for managing reproductive health and optimizing assisted reproduction in DENV exposed patients, crucial for minimizing the risks that have already been related.
